# Waiting time to see a dermatologist in South Africa: A cross-sectional survey

**DOI:** 10.4102/jcmsa.v4i1.359

**Published:** 2026-03-16

**Authors:** Ruan S. de Jager, Nicola A. Gray, Willem I. Visser

**Affiliations:** 1 Division of Dermatology, Faculty of Medicine and Health Sciences, Stellenbosch University, Cape Town, South Africa; 2 Department of Dermatology, Tygerberg Hospital, Cape Town, South Africa; 3 Department of Dermatology and Skin Science, Faculty of Medicine, University of British Columbia, Vancouver, Canada

**Keywords:** dermatology, waiting times, healthcare access, geographic disparities, private healthcare

## Abstract

**Background:**

Waiting times for specialist consultations influence timely diagnosis and appropriate disease management. Given the high prevalence and morbidity associated with skin diseases, prolonged waiting times to access dermatological care have important public health implications. In South Africa, data describing waiting times for dermatology services are lacking.

**Methods:**

This descriptive cross-sectional study assessed waiting times for private-sector dermatology services across South Africa. All dermatology practices listed on Medpages were contacted telephonically on three separate occasions between 01 and 31 August 2023. Using a standardised script, appointment availability was requested for a fictitious patient under three clinical scenarios: a routine skin check, a changing pigmented lesion suggestive of possible melanoma and a cosmetic consultation for neurotoxin injection.

**Results:**

A total of 192 dermatologists were included. The national median waiting time was 15 days (interquartile ranges [IQR] 5–48.3 days) for a routine skin check, 5 days (IQR 1–14 days) for suspected melanoma and 12 days (IQR 4–33.8 days) for cosmetic consultation. Waiting times varied widely across provinces, ranging from same-day access to delays exceeding 1 year. The longest median waiting time for routine consultations was observed in the Northern Cape (227 days), while the shortest was in Limpopo (2 days). Consultation fees also showed substantial interprovincial variation.

**Conclusion:**

While overall waiting times appear acceptable at a national level, marked provincial disparities exist. Urgent cases are generally prioritised, yet prolonged delays in certain regions may compromise timely care, underscoring the need for targeted, context-specific interventions.

**Contribution:**

This study provides the first national overview of private-sector dermatology waiting times in South Africa. It highlights significant geographic disparities in access to care within the private healthcare system and generates evidence to inform health service planning, workforce distribution and strategies to improve equitable access to dermatological services.

## Introduction

Skin diseases are often underestimated yet constitute a substantial component of the global burden of disease. They are among the most prevalent medical conditions worldwide and are frequently associated with significant morbidity, either through visible disfigurement or persistent, distressing symptoms such as chronic pruritus.^1^ Across diverse geographic and socioeconomic settings, multiple authors have highlighted a mismatch between the high burden of skin disease and the limited availability of trained dermatologists.^2,3^

South Africa, classified as a low- to middle-income country, faces particular challenges in delivering adequate dermatological services. These challenges are compounded by a marked shortage of dermatologists relative to the size and needs of the population.^4^

Waiting times for specialist care are widely recognised as important indicators of healthcare access, system efficiency and service quality.^5^ Prolonged waiting times may delay diagnosis and initiation of appropriate treatment, potentially leading to disease progression, avoidable complications and poorer patient outcomes. Extended delays are also associated with increased patient dissatisfaction and reduced confidence in healthcare services.^6^ While waiting times for dermatology consultations have been investigated in several high-income and selected middle-income countries, there are currently no published data describing dermatology waiting times in South Africa.^7^

In a resource-constrained healthcare system characterised by high dermatological disease burden and limited specialist capacity, an understanding of waiting times is essential to identify gaps in service delivery and inequities in access to care. This study aims to determine waiting times for dermatology appointments in South Africa. By evaluating waiting times across different clinical indications and geographical regions within the private healthcare sector, this research seeks to generate data that may inform health service planning and contribute to strategies aimed at improving equitable access to dermatological care.

## Research methods and design

### Study design and setting

This descriptive cross-sectional study was conducted within the private healthcare sector across all nine provinces of South Africa.

### Study objectives

The primary objective was to determine the average waiting time to obtain an appointment with a specialist dermatologist under three predefined clinical scenarios: (1) a routine dermatology consultation, (2) an urgent consultation for a changing pigmented lesion suggestive of possible melanoma, and (3) a cosmetic consultation for neurotoxin injection. Secondary objectives included comparison of waiting times across provinces, determination of consultation fees for routine visits nationally and by province and assessment of the proportion of practices willing to submit claims to medical insurers on behalf of patients.

### Study population and sampling

All medical practitioners registered with the Health Professions Council of South Africa (HPCSA) as specialist dermatologists and practising in the private sector were eligible for inclusion. Neither the HPCSA nor the Colleges of Medicine of South Africa were able to provide a comprehensive database of private sector dermatologists. Participants were therefore identified using Medpages,^8^ the largest online directory of private medical practitioners in South Africa, where clinicians are listed by speciality.

General practitioners with a special interest in dermatology (so-called GP dermatologists) were excluded. Dermatologists practising exclusively in the public healthcare sector were also excluded, as they cannot be contacted directly to arrange private consultations. Only in-person consultations were considered; teledermatology services were excluded.

### Data collection

Practice telephone numbers listed on Medpages were contacted telephonically by the investigator on three separate occasions to enquire about appointment availability for each of the three predefined scenarios. A standardised script was used for all calls. For each scenario, the earliest available appointment date was recorded; no appointments were confirmed or booked.

Calls were conducted on three separate weekdays between 01 August 2023 and 31 August 2023. Waiting time was calculated as the number of days between the date of the telephone call (day 0) and the proposed appointment date. Where more than one dermatologist practised at the same location, the earliest available appointment at that practice was recorded. Practices that could not be reached telephonically on three separate occasions were excluded.

### Statistical analysis

Continuous variables were summarised using means with standard deviations (s.d.) for normally distributed data and medians with interquartile ranges (IQR) for non-normally distributed data. Data analysis was performed using Microsoft^®^ Excel^®^ (Version 2403). Spearman’s rank correlation coefficient was used to assess the strength of association for non-normally distributed variables and was calculated using the RANK.AVG and CORREL functions.^9^ Correlation coefficients were interpreted as follows: ± 0.90–1.00 very high, ± 0.70–0.89 high, ± 0.50–0.69 moderate, ± 0.30–0.49 low and ± 0.29–0.00 negligible.^10^

### Ethical considerations

Ethical approval for the study was obtained from Stellenbosch University Health Research Ethics Committee (Reference number: S23/05/109). This study did not involve direct interaction with patients, access to patient records or the collection of personal health information. Practice staff were contacted telephonically using publicly available contact details to enquire about appointment availability. As no identifiable personal data were collected and no appointments were confirmed, the study was classified as minimal-risk research. Formal written or oral consent was therefore not required.

To maintain confidentiality, no personal identifiers of healthcare practitioners or practice staff were recorded. Data were anonymised at the point of collection, stored securely and analysed in aggregate form only.

## Results

### Study participants

A total of 246 dermatologists were identified on Medpages. Of these, 30 practised exclusively in the public sector and were excluded. Thirteen dermatologists could not be contacted on three separate occasions. One dermatologist was on maternity leave, one was on sabbatical and one had recently retired. Two dermatologists accepted referrals exclusively for Mohs micrographic surgery, one offered only cosmetic consultations, one accepted referral solely for laser treatments, and six were not accepting new patients at the time of contact. After exclusions, 192 dermatologists were included in the final analysis.

### Geographic distribution of dermatologists

**FIGURE 1:**
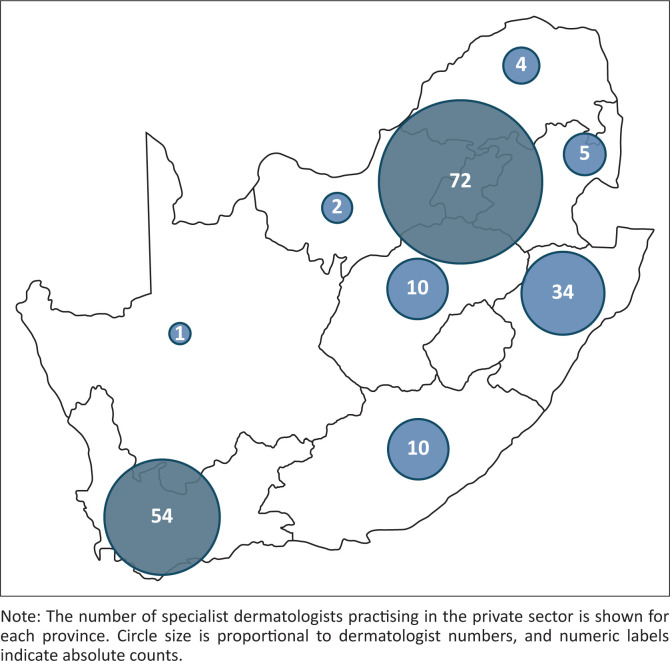
Provincial distribution of private-sector dermatologists in South Africa.

The provincial distribution of included dermatologists is presented in Table 1 and illustrated in Figure 1. Most dermatologists were located in Gauteng (37.5%), followed by the Western Cape (28.1%) and KwaZulu-Natal (17.7%). Smaller proportions were found in the Eastern Cape (5.2%), Free State (5.2%), Mpumalanga (2.6%), Limpopo (2.1%), North West (1.0%), and Northern Cape (0.5%).

**TABLE 1:** Number of dermatologists per province.

Province	*n*	%
Gauteng	72	37.5
Western Cape	54	28.1
KwaZulu-Natal	34	17.7
Eastern Cape	10	5.2
Free State	10	5.2
Mpumalanga	5	2.6
Limpopo	4	2.1
North West	2	1.0
Northern Cape	1	0.5

**Total**	**192**	**100**

### Waiting times for dermatology consultations

**FIGURE 2:**
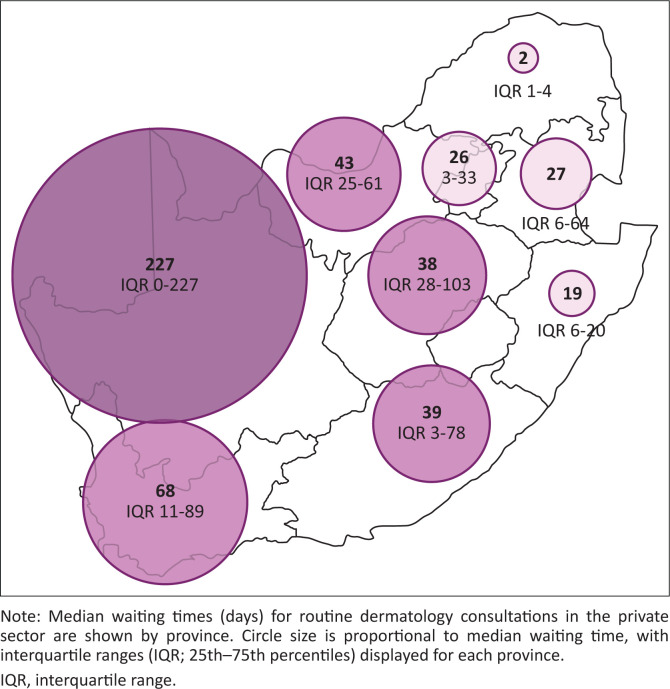
Provincial variation in waiting times for routine skin evaluation.

**FIGURE 3:**
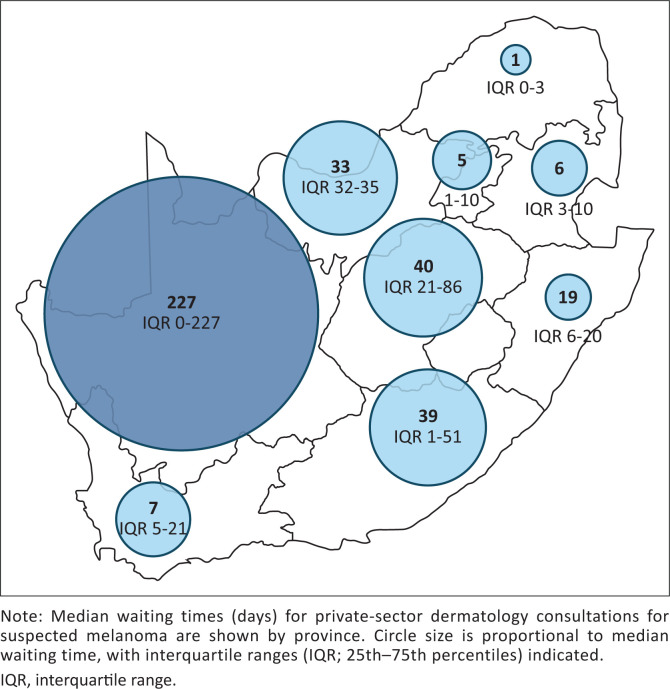
Provincial variation in waiting times for suspected melanoma consultations.

**FIGURE 4:**
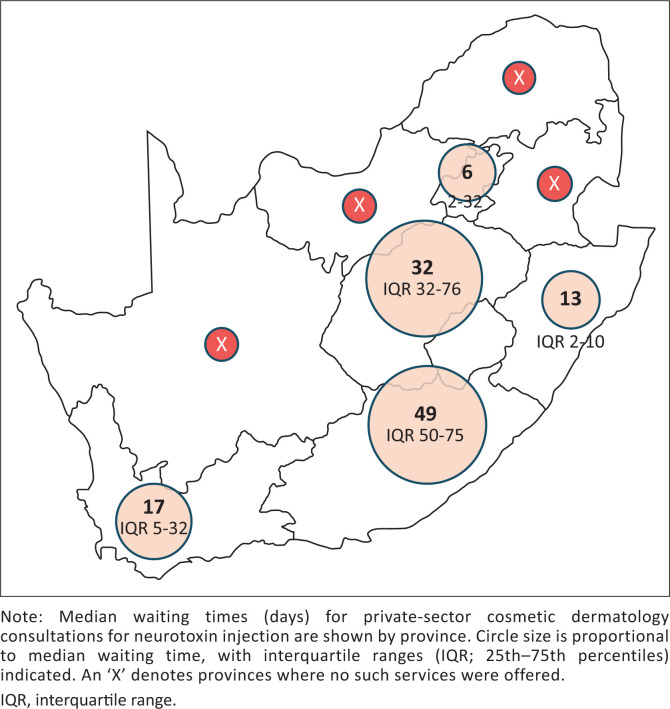
Provincial variation in waiting times for cosmetic neurotoxin consultations.

Median waiting times for dermatology appointments varied widely both nationally and between provinces (Table 2), as illustrated in Figure 2. Nationally, the median waiting time was 15 days (IQR 5–48.3 days) for a routine skin check, 5 days (IQR 1–14 days) for a consultation for a changing pigmented lesion suggestive of possible melanoma and 12 days (IQR 4–33.8 days) for a cosmetic consultation. Provincial variation in waiting times for consultations for suspected melanoma and cosmetic neurotoxin consultations is shown in Figure 3 and Figure 4, respectively.

**TABLE 2:** Median waiting times to see a private dermatologist by province in South Africa (days).

Province	Number of practices	Routine skin evaluation	IQR	Neurotoxin injections	IQR	Possible melanoma	IQR
Northern Cape	1	227.0	227.0	-	-	-	-
North West	2	43.0	25.0–61.0	-	-	33.5	32.3–34.8
Eastern Cape	10	39.0	2.5–77.8	70.0	49.5–75.3	2.0	1.0–51.0
Free State	10	38.5	28.3–103.8	39.0	32.0–76.0	41.0	21.3–86.0
Mpumalanga	5	27.0	6.0–64.0	-	-	6.0	2.8–10.0
Western Cape	54	20.5	11.0–89.0	17.0	5.0–31.5	7.0	5.0–21.0
KwaZulu-Natal	34	11.5	6.0–20.5	13.0	8.0–18.0	2.5.0	2.0–10.0
Gauteng	72	7.5	3.0–33.0	6.5	2.0–32.0	5.0	1.0–10.0
Limpopo	4	2.0	1.0–3.8	-	-	1.5	0.0–3.3
**South Africa**	**192**	**15.0**	**5.0–48.3**	**12.0**	**4.0–33.8**	**5.0**	**1.0–14.0**

IQR, interquartile range.

For routine skin checks, waiting times across individual practices ranged from same-day availability (0 days) to a maximum of 421 days. The provinces with the longest median waiting times for routine consultations were the Northern Cape (227 days), North West (43 days) and Eastern Cape (39 days). The shortest median waiting times were observed in Limpopo (2 days), Gauteng (7.5 days), and KwaZulu-Natal (11.4 days).

In 10 instances where the script for a changing pigmented lesion was used, practices with very long waiting times declined to provide an appointment date and instead advised consultation with a general practitioner, who could refer the patient urgently if deemed necessary. As no appointment dates were provided, these cases were excluded from the waiting-time analysis. These practices were located in the Free State (*n* = 2), Gauteng (*n* = 1), Northern Cape (*n* = 1), Western Cape (*n* = 3), KwaZulu-Natal (*n* = 2), and Mpumalanga (*n* = 1).

A strong negative correlation was observed between provincial contribution to gross domestic product (GDP) and median waiting times for routine skin checks (Spearman’s ρ = −0.78). In contrast, the correlation between the number of dermatologists per million population^11^ and routine waiting times was negligible (Spearman’s ρ = −0.13).

### Consultation fees

The national median consultation fee for a routine dermatology visit in private practice was R1050.00 (IQR R900.00–R1263.00). Median consultation fees were highest in the Western Cape (R1250.00) and lowest in the Northern Cape (R800.00) (Table 3). There was a strong positive correlation between provincial GDP contribution and consultation fees (Spearman’s ρ = 0.82).

**TABLE 3:** Consultation fees for private dermatology consultations by province in South Africa (South African Rand).

Region	Number of practices	Median	IQR	Min.	Max.
Western Cape	53	1250	1050–1360	750	1600
Gauteng	72	1075	900–1281	750	1650
Mpumalanga	5	1050	1000–1100	750	1200
Eastern Cape	10	950	825–973	750	1200
KwaZulu-Natal	34	925	890–1200	800	2300
Limpopo	4	875	838–913	800	950
Free State	10	835	763–850	600	1800
North West	2	825	-	700	950
Northern Cape	1	800	-	-	-
**South Africa**	**191**	**1050**	**900–1263**	**600**	**2300**

IQR, interquartile range; Min., minimum; Max., maximum.

Most private dermatology practices required direct payment from patients at the time of consultation. Of the practices contacted, 55.2% (*n* = 106) did not submit claims to medical insurers on behalf of patients, while 41.7% (*n* = 80) did submit claims directly. A small proportion (2.6%, *n* = 5) were willing to submit claims only for selected medical aid schemes. Billing information was not obtained from one practice.

### Availability of cosmetic services

Eighty-five practices (44.3%) offered cosmetic consultations for neurotoxin injections, while 107 practices (55.7%) did not. No cosmetic consultation services were offered by dermatology practices in Limpopo, Mpumalanga, Northern Cape, or North West.

## Discussion

The most striking finding of this study is that, while overall median waiting times for private dermatology consultations in South Africa appear acceptable, there is marked variation between provinces. Though inequality between the public and private healthcare sectors in South Africa is well recognised, this study highlights substantial inequity *within* the private sector itself, driven by geographic location. Important secondary findings include significant variation in consultation fees and the observation that only a minority of dermatologists in South Africa offer cosmetic services.

### Waiting times in South Africa’s private healthcare sector

The median waiting time to see a dermatologist in South Africa’s private sector was 15 days (IQR 5–48.3 days) for a routine skin check, 5 days (IQR 1–14 days) for a suspected melanoma and 12 days (IQR 4–33.8 days) for a cosmetic consultation. When placed in an international context, these waiting times compare favourably. In the United States, average waiting times for new dermatology appointments are approximately 36 days, with reported ranges from 9 days to 120 days depending on the state.^12^ In Brazil, private-sector dermatology waiting times range between 2 and 15 working days, with regional variability.^13^

The relatively short overall waiting times observed in South Africa’s private sector are likely attributable to the higher concentration of dermatologists serving a comparatively small, insured population. According to Tiwari et al., South Africa has approximately 1.2 dermatologists per million population in the public sector compared with 20.1 per million in the private sector.^11^ Despite this disparity, both sectors remain under-resourced when compared with international benchmarks, with the World Health Organization suggesting an ideal ratio of approximately four dermatologists per 100 000 population.^14^

### Provincial variation in waiting times

Substantial interprovincial differences were observed for routine dermatology consultations. The longest median waiting times were recorded in the Northern Cape (227 days), North West (43 days) and Eastern Cape (39 days), while the shortest were observed in Limpopo (2 days), Gauteng (7.5 days), and KwaZulu-Natal (11.4 days). Notably, a strong negative correlation was identified between provincial contribution to GDP and waiting times, with wealthier provinces experiencing shorter delays.

This association likely reflects the clustering of private dermatology services in economically stronger regions that offer greater professional and personal opportunities. The Northern Cape illustrates this disparity starkly: despite covering a vast geographic area, it contributes only 2.2% to national GDP and is served by a single private-sector dermatologist, resulting in the longest waiting times observed in this study. Similar associations between socioeconomic factors and access to specialist care have been reported internationally.^15^

In contrast, no meaningful correlation was observed between the number of dermatologists per million population and waiting times. This may reflect the fact that only a minority of South Africans access private healthcare, rendering total provincial population figures a poor proxy for demand. Limpopo, for example, is the fifth most populous province yet has only four private dermatologists and the shortest waiting times, likely due to its low medical aid coverage (9.5%).^16^ This finding aligns with international literature demonstrating that specialist density alone does not necessarily translate into improved access, though the underlying drivers differ between healthcare systems.^13,17^

### Urgent referrals versus cosmetic consultations

Private dermatology practices appear to prioritise urgent over routine consultations. International melanoma guidelines recommend that patients with suspected melanoma be assessed by an appropriate healthcare professional within 2 weeks of suspicion.^18^ In this study, patients with a suspected melanoma were generally seen more rapidly than those seeking routine or cosmetic care. However, only Mpumalanga, Limpopo, KwaZulu-Natal, and Gauteng consistently met this 2-week benchmark.

Of concern, 18 practices offered appointment dates exceeding 60 days for suspected melanoma without advising alternative or interim medical assessment. Delays in melanoma diagnosis and definitive treatment are associated with increased morbidity and mortality,^19,20^ underscoring the clinical implications of these findings. The Northern Cape practice was unable to offer an appointment and appropriately advised urgent assessment by a general practitioner with onward referral if required.

The Free State was the only province where cosmetic consultations were, on average, available sooner than consultations for suspected melanoma. This inversion of clinical priority is concerning and highlights the need for clearer triage pathways within private practices.

In contrast to countries such as Canada, where specialist access requires referral from a primary care provider,^3^ patients in South Africa’s private sector may self-refer. While this improves access, it may also result in specialist appointments being occupied by conditions manageable at primary care level, potentially contributing to extended waiting times for more urgent cases.

### Cost of care and insurance coverage

Most private dermatology practices (58.3%) required direct patient payment and did not submit claims to medical insurers. This may reflect administrative burden, delayed reimbursement or discrepancies between insurer tariffs and actual consultation fees. Medical aid reimbursement rates are often substantially lower than the median consultation fee of R1095.00, potentially creating further barriers to access.

Consultation fees broadly reflected provincial economic status, with the highest median fees observed in the Western Cape, Gauteng, and KwaZulu-Natal, and the lowest in Limpopo, Northern Cape and North West. Mpumalanga was a notable exception, demonstrating relatively high consultation fees despite a lower provincial GDP.^21^

### Cosmetic dermatology services

Only 44.7% of dermatologists in this study offered cosmetic procedures, and no cosmetic services were available in four provinces. This is notable given the global expansion of medical aesthetic procedures, including neurotoxin injections and dermal fillers, over the past two decades.^22^

Cosmetic dermatology is not formally included in the College of Medicine of South Africa’s dermatology curriculum,^23^ which may contribute to variable confidence and uptake among specialists. Furthermore, cosmetic procedures in South Africa are frequently provided by general practitioners, dentists and plastic surgeons. In the context of limited specialist capacity and a high burden of medical and surgical dermatological disease, the role of dermatologists in cosmetic medicine remains contested.

### Limitations and future directions

This cross-sectional study captured dermatology waiting times during a single time period (August 2023) and therefore does not reflect temporal variation or trends over time. Waiting times may also have been influenced by residual effects of the coronavirus disease 2019 (COVID-19) pandemic on healthcare delivery and workforce availability. Longitudinal studies would be better suited to evaluate changes in waiting times over time and to corroborate the findings of this study.

Identification of dermatology practices relied on Medpages, which lists most private-sector dermatologists in South Africa but includes only those practitioners who have consented to share their contact details. It is therefore possible that some eligible practices were not captured, which may have resulted in under-representation in certain regions.

This study was limited to the private healthcare sector. As the majority of South Africans receive care within the public healthcare system, these findings cannot be generalised to dermatology services nationally. Future research should assess waiting times in the public sector and explore disparities between public and private dermatology services. In addition, qualitative studies examining patient experiences and perceptions of waiting times would provide valuable context to the quantitative findings.

Teledermatology services were not assessed in this study. The inclusion of virtual dermatology consultations may influence access to care and reduce waiting times, particularly in underserved or geographically remote areas. International evidence suggests that teledermatology can reduce unnecessary in-person consultations and improve cost-effectiveness,^24^ and its role within the South African context warrants further investigation.

## Conclusion

This study demonstrates that, while urgent dermatological conditions are generally prioritised over routine and cosmetic consultations in South Africa’s private healthcare sector, access to care varies markedly by geographic location. Across all provinces, waiting times for dermatology consultations showed substantial variability, ranging from same-day access to delays exceeding 1 year. A key finding is the strong association between provincial economic strength and waiting times, with provinces contributing less to national GDP experiencing longer delays.

These disparities highlight inequities within the private healthcare sector itself, beyond the well-recognised divide between public and private care. Though overall median waiting times appear acceptable at a national level, prolonged delays in certain provinces raise concerns regarding timely diagnosis and management, particularly for potentially life-threatening conditions such as melanoma.

The findings underscore the need for targeted, context-specific strategies to improve equitable access to dermatological care, including workforce distribution, service organisation and referral pathways. Future research should incorporate longitudinal designs, public-sector analyses and qualitative assessments of patient experiences to better inform policy development and health system planning aimed at strengthening dermatology services and reducing geographic inequities in South Africa.
